# Software codesign between end users and developers to enhance utility for biodiversity conservation

**DOI:** 10.1093/biosci/biae097

**Published:** 2024-10-22

**Authors:** Mary E Blair, Elkin A Noguera-Urbano, Jose Manuel Ochoa-Quintero, Andrea Paz, Cristina Lopez-Gallego, María Ángela Echeverry-Galvis, Juan Zuloaga, Pilar Rodríguez, Leonardo Lemus-Mejia, Peter Ersts, Daniel F López-Lozano, Matthew E Aiello-Lammens, Hector M Arango, Leonardo Buitrago, Samuel Chang Triguero, Cristian A Cruz-Rodríguez, Juan F Díaz-Nieto, Dairo Escobar, Valentina Grisales-Betancur, Bethany A Johnson, Jamie M Kass, María C Londoño-Murcia, Cory Merow, Carlos J Muñoz-Rodríguez, María Helena Olaya-Rodríguez, Juan L Parra, Gonzalo E Pinilla-Buitrago, Nicolette S Roach, Octavio Rojas-Soto, Néstor Roncancio-Duque, Erika Suárez-Valencia, J Nicolás Urbina-Cardona, Jorge Velásquez-Tibatá, Camilo A Zapata-Martinez, Robert P Anderson

**Affiliations:** Center for Biodiversity and Conservation, American Museum of Natural History, New York, New York, United States; Instituto de Investigación de Recursos Biológicos Alexander von Humboldt, Bogotá, Distrito Capital, Colombia; Instituto de Investigación de Recursos Biológicos Alexander von Humboldt, Bogotá, Distrito Capital, Colombia; Department of Environmental Systems Science, Institute of Integrative Biology, Zürich, Switzerland; Instituto de Biología, Universidad de Antioquia, Medellín, Colombia; Departamento de Ecología y Territorio, Facultad de Estudios Ambientales y Rurales, Pontificia Universidad Javeriana, Bogotá, Distrito Capital, Colombia; Department of Biology, McGill University, Montréal, Québec, Canada; Comisión Nacional para el Conocimiento y Uso de la Biodiversidad, Ciudad de México, México; ProCat Colombia, Bogotá, Distrito Capital, Colombia; Center for Biodiversity and Conservation, American Museum of Natural History, New York, New York, United States; Center for Biodiversity and Conservation, American Museum of Natural History, New York, New York, United States; Instituto de Investigación de Recursos Biológicos Alexander von Humboldt, Bogotá, Distrito Capital, Colombia; Department of Environmental Studies and Science, Pace University, Pleasantville, New York, United States; Instituto de Investigación de Recursos Biológicos Alexander von Humboldt, Bogotá, Distrito Capital, Colombia; Latin America and Caribbean Regional Support, Global Biodiversity Information Facility; Universidad Nacional de Colombia, Bogotá, Distrito Capital, Colombia; Department of Environmental Studies and Science, Pace University, Pleasantville, New York, United States; Instituto de Investigación de Recursos Biológicos Alexander von Humboldt, Bogotá, Distrito Capital, Colombia; Département de Sciences Biologiques, Université de Montréal, Montréal, Québec, Canada; Natural Systems and Sustainability Area, Universidad EAFIT, Medellín, Colombia; Bogotá, Distrito Capital, Colombia; Natural Systems and Sustainability Area, Universidad EAFIT, Medellín, Colombia; El Globo Nature Reserve, Támesis, Colombia; Center for Biodiversity and Conservation, American Museum of Natural History, New York, New York, United States; Department of Biology, City College of New York, City University of New York, New York, New York, United States; Macroecology Laboratory, Graduate School of Life Sciences, Tohoku University, Sendai, Miyagi, Japan; Instituto de Investigación de Recursos Biológicos Alexander von Humboldt, Bogotá, Distrito Capital, Colombia; University of Connecticut, Storrs, Connecticut, United States; Instituto de Investigación de Recursos Biológicos Alexander von Humboldt, Bogotá, Distrito Capital, Colombia; Instituto de Investigación de Recursos Biológicos Alexander von Humboldt, Bogotá, Distrito Capital, Colombia; Instituto de Biología, Universidad de Antioquia, Medellín, Colombia; Department of Biology, City College of New York, City University of New York, New York, New York, United States; Graduate Center of the City University of New York, New York, New York, United States; Department of Ecology and Conservation Biology, Texas A&M University, College Station, Texas, United States; Instituto de Ecología AC, Veracruz, México; Parque Nacional Natural Las Hermosas, Palmira; Instituto Amazónico de Investigaciones Científicas—Sinchi, Inírida, Colombia; Instituto de Investigación de Recursos Biológicos Alexander von Humboldt, Bogotá, Distrito Capital, Colombia; Departamento de Ecología y Territorio, Facultad de Estudios Ambientales y Rurales, Pontificia Universidad Javeriana, Bogotá, Distrito Capital, Colombia; Audubon Americas, National Audubon Society, Bogotá, Distrito Capital, Colombia; Instituto de Investigación de Recursos Biológicos Alexander von Humboldt, Bogotá, Distrito Capital, Colombia; Department of Biology, City College of New York, City University of New York, New York, New York, United States; Graduate Center of the City University of New York, New York, New York, United States; Division of Vertebrate Zoology, American Museum of Natural History, New York, New York, United States

**Keywords:** applied ecology, biodiversity, biogeography, conservation, informatics

## Abstract

Creating software tools that address the needs of a wide range of decision-makers requires the inclusion of differing perspectives throughout the development process. Software tools for biodiversity conservation often fall short in this regard, partly because broad decision-maker needs may exceed the toolkits of single research groups or even institutions. We show that participatory, collaborative codesign enhances the utility of software tools for better decision-making in biodiversity conservation planning, as demonstrated by our experiences developing a set of integrated tools in Colombia. Specifically, we undertook an interdisciplinary, multi-institutional collaboration of ecological modelers, software engineers, and a diverse profile of potential end users, including decision-makers, conservation practitioners, and biodiversity experts. We leveraged and modified common paradigms of software production, including codesign and agile development, to facilitate collaboration through all stages (including conceptualization, development, testing, and feedback) to ensure the accessibility and applicability of the new tools to inform decision-making for biodiversity conservation planning.

Nimble policy responses to conserve biodiversity, such as to meet the 2030 Kunming–Montreal Global Biodiversity Framework targets, are only possible with information from adaptable analytic frameworks that link multiple data sources (Gonzalez and Londoño 2022). Such information is essential to global assessments of biodiversity, such as those led by the IPBES (Intergovernmental Science-Policy Platform on Biodiversity and Ecosystem Services) and the IUCN (International Union for the Conservation of Nature), and also as input to inform decision-makers at various scales. Toward these ends, biodiversity observation networks (Gonzalez et al. 2023) obtain, funnel, and facilitate the near real-time analysis of field-derived data and remotely sensed observations to quantify the status of biodiversity and predict scenarios for its future.

More generally, creating software tools that address the needs of a wide range of decision-makers requires the inclusion of differing perspectives throughout the development process. Software tools for biodiversity conservation often fall short in this regard, partly because the needs of broad decision-makers may exceed the toolkit capability of single research groups or even institutions. In the present article, we show that participatory, collaborative codesign across many institutions enhances the utility of software tools for better decision-making in biodiversity conservation planning, as was demonstrated by our experiences developing a set of integrated tools in Colombia. Specifically, we are a team of ecological modelers, software engineers, biodiversity experts, and conservation practitioners from a wide range of disciplines, backgrounds, and institutions who came together to serve the needs of the Colombia Biodiversity Observation Network (https://geobon.org/bons/national-regional-bon/national-bon/colombia-bon). Our authorship includes both end users and developers with a broad range of skills and knowledge, brought together with the goal of leveraging and modifying common paradigms of software production, including codesign and agile development (Hohl et al. 2018). During this process, we facilitated collaboration and inclusion through all stages of our efforts (including conceptualization, development, testing, and feedback) to ensure the accessibility, applicability, and impact of the new tools.

## Software codesign for biodiversity conservation planning

Our experiences derive from activities under the Group on Earth Observations Biodiversity Observation Network (GEO BON). GEO BON facilitates the development of national, regional, and thematic biodiversity observation networks (Gonzalez et al. 2023) and related software tools to connect end users and software tool developers worldwide. Importantly, GEO BON standardized a series of essential biodiversity variables to harmonize monitoring by leveraging remote sensing data sets (Pereira et al. 2013). Strategic funding initiatives to connect essential biodiversity variables with decision-making, such as the NASA program that funded our efforts, have promoted the development of decision-support systems: software tools that aim to track essential biodiversity variables to support specific decision-making applications (GEO BON 2017).

Species distribution models (SDMs) estimate environmental suitability and are useful for estimating the essential biodiversity variable ‘species distribution’ (Pereira et al. 2013, Vihervaara et al. 2017, Urbina-Cardona et al. 2019). SDMs harness biodiversity data (occurrence records of a species) and can guide conservation decision-making, especially when they integrate expert knowledge (Araújo et al. 2019, Velásquez-Tibatá et al. 2019, Merow et al. 2022). Postprocessing SDMs can increase their utility for biodiversity conservation decision-making by incorporating current remote sensing and other information reflecting land use, overexploitation, or additional factors (Merow et al. 2022). They can also be transferred across space and time to forecast invasions or changes in biodiversity under dynamic climatic and land-use scenarios. However, until recently, building high-quality SDMs involved a steep learning curve, and their use in decision-support systems had been hindered by the lack of didactic, user-friendly software tools and frameworks for expert vetting of data inputs, as well as for building and postprocessing SDMs (Gonzalez and Londoño 2022).

In the present article, we leveraged two innovative tools and brought together a broad set of end users and developers to cocreate software that meets the needs for quantifying the essential biodiversity variable ‘species distribution’. Critically, the Colombia Biodiversity Observation Network (Gonzalez et al. 2023) builds and maintains a vibrant community to develop biodiversity information systems at national and subnational scales. To promote biodiversity change indicator assessments, we partnered with the Colombia Biodiversity Observation Network to enhance the open-source, user-friendly, and modular SDM-building application Wallace EcoMod (Kass et al. 2018, 2023; hereafter, Wallace). Together, we identified the need to achieve interoperability between Wallace and BioModelos, the Colombia Biodiversity Observation Network’s existing server-based application for expert vetting of species occurrence data sets and hosted models of their distributions (Velásquez-Tibatá et al. 2019). Synergistically, these connections would allow users to upload vetted occurrence data from BioModelos into Wallace and send range estimates from postprocessed SDMs produced in Wallace back to BioModelos. To achieve this, we assembled a diverse group to codesign and develop software packages that leverage remote sensing data to postprocess SDM outputs in order to estimate species’ current ranges and calculate indicators of biodiversity change: *maskRangeR* (Merow et al. 2022) and *changeRangeR* (Galante et al. 2023). We then integrated these packages as new modules in Wallace, enabling reports on the status, trends, and drivers of biodiversity changes, which are all informed by accessible, documented, and reproducible analyses (https://github.com/wallaceEcoMod/wallace/tree/biomodelos). In addition, we enabled data publication (push) and retrieval (pull) from and to the BioModelos API (application programming interface).

**Figure 1.**
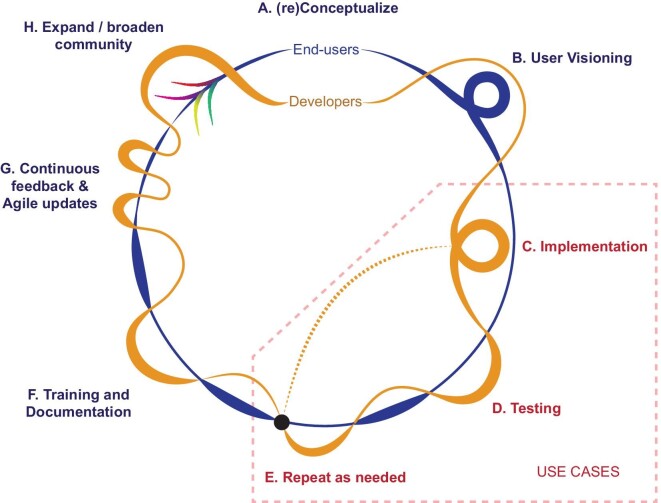
Steps in our flexible implementation of the core steps of agile software development to achieve codesign of extensions of Wallace EcoMod and BioModelos for biodiversity conservation users. Software developers (the orange line) and end users (the blue line) iteratively interact (see the line intersections) and engage more intensely (see the loops) or exchange leadership of engagements (the thicker lines) during the process, showing more flexibility in how and when end users are engaged than in typical agile frameworks. In addition, we found that only certain elements specified in typical agile software development methodologies were necessary, especially those related to regular inclusion of and communication with users (steps B, D, and G). In contrast, following a formalized agile framework (such as scrum, with concerted sprints for development of particular tasks) was not needed. The wisps in step H represent how the software can have utility for additional users by being included in a subsequent round of development.

Throughout the iterative codesign, development, and testing process, software developers engaged with end users (figure 1). Merging aspects of codesign and agile development paradigms, this process included cycles of engagement activities where users make their needs and goals explicit—for example, user mapping and visioning, alpha and beta releases and testing, and integration of agile updates (Brown 2008, Hohl et al. 2018). To enhance the potential for broad accessibility, applicability, and impact of the new tool, the team included end users from a wide variety of sectors and organizations (e.g., nongovernmental, governmental, academic) with different kinds of management decisions they wanted to inform (e.g., protected-area planning, monitoring, environmental-impact offsets).

## Examples of use

As a first example of a user context that informed the codesign (figure 1, step A), our team included practitioners responsible for national and global assessments of species extinction risk (e.g., global and national red lists). Extinction risk assessments such as those of the IUCN are ideally done with information on population size, geographic distribution, and associated temporal trends (IUCN 2024). However, in most cases, only rough information regarding current species distributions is available (Anderson 2023). In contrast, SDMs can provide information on both the current and potential future distributions of species, and remote sensing products can then be harnessed in postprocessing to quantify temporal trends, such as recent habitat loss and extinction risk. Although some existing software packages provide relevant tools to calculate spatial metrics such as the IUCN area of occupancy (e.g., *conR*), analyses of the impacts of particular threats are usually only qualitative. The group saw that such analyses could be quantified and compared over time using remote sensing products, if a tool were created in a way to be accessible to practitioners.

**Figure 2.**
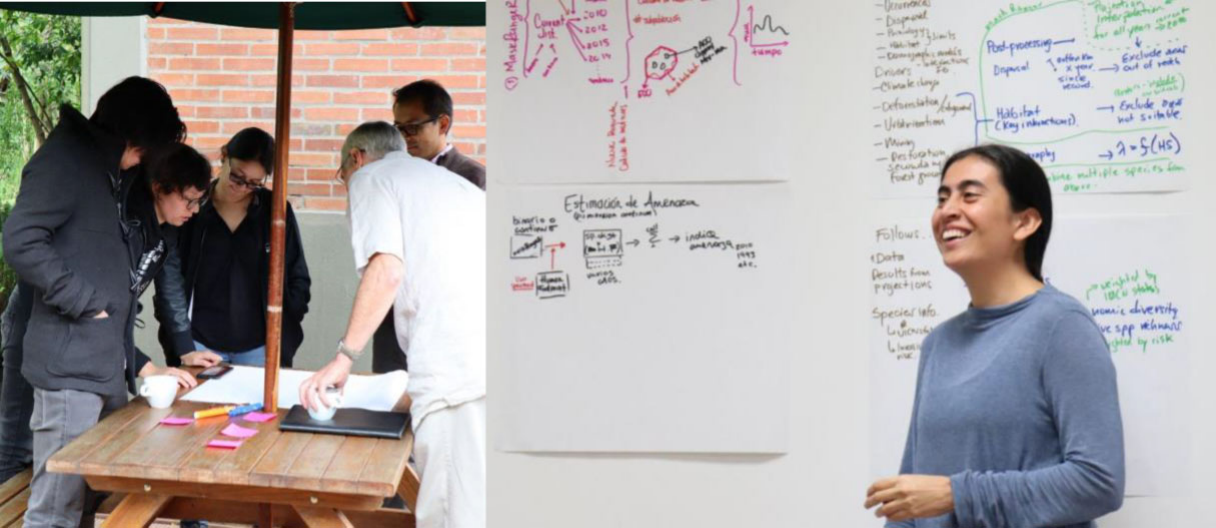
User-visioning exercise (left) and discussion (right) during a user consultation workshop in Bogotá, Colombia, in 2019.

Through user-visioning exercises (figure 2, box 1), we explored options for enhancing Wallace in a way that could harness SDMs to contribute to extinction-risk assessments and conservation planning (figure 1, step B). We designed a postprocessing workflow to combine SDMs with information on habitat quantity and quality from remote sensing and derived human-footprint layers (Correa Ayram et al. 2020) to inform red-list assessments and weigh the potential impacts of specific threats (e.g., habitat degradation) and conservation actions (e.g., coverage in protected areas). From this vision, we developed the R packages *maskRangeR* and *changeRangeR* and added them to Wallace (figure 1, step C). The packages and their inclusion into Wallace were tested with several rounds of feedback and adjustments (figure 1, steps D and E) to estimate red-list metrics using SDMs and quantify some aspects of uncertainty. In total, after the initial in-person workshop for visioning, we convened two additional ones (one in person and one virtual) largely focused on testing new features and gathering feedback from users to make further improvements. The development team also circulated an online testing and feedback form to reach a broader group of users, as well as held at least 10 additional smaller meetings with specific end users for detailed feedback on specific features, needs, and issues around implementation and use cases.

Ultimately, the new packages and their integration into Wallace allowed users to standardize their analyses regarding the effects of threats and conservation actions on species distributions by providing quantitative and spatially explicit information. This resulted in key information toward assessments that supported the National List of Threatened Wild Species of Colombia (MADS 2024), including assessments for plants (Lopez-Gallego and Morales-Morales 2023), primates (Henao-Díaz et al. 2020), rodents (Ramírez-Chaves et al. 2022), and birds (Chaparro-Herrera et al. 2024), supporting the appropriate allocation of conservation responsibilities to local authorities based on expert-vetted spatially explicit information on threatened species’ distributions.

Although the red-list context informed the initial development cycle, our diverse group also included users whose needs guided further enhancements for broader use and applicability to a wider range of conservation decision-making contexts. This was facilitated by developing additional case studies (use cases; figure 1) and running multiple iterations of the whole cycle after broadening community involvement (figure 1, step H). In one additional user context that informed the codesign, researchers wanted to estimate and map species richness, endemism, and functional diversity to improve the selection of conservation-planning strategies under climate change. They had explored other tools for this purpose but halted their efforts because of steep learning curves and computational demands. With the specific goal of comparing the potential distributions of 135 mammal species under current

Box 1. **User-visioning exercises.**

User-visioning exercises such as story mapping are often employed in user-centered software design approaches, such as agile software development. We took a highly participatory approach to implementing these exercises. In a multiday user consultation workshop, the coordinators engaged conservation practitioners and biodiversity experts in two facilitated exercises to generate, present, and prioritize visions for the software being developed with their needs in mind. First, in small groups, participants wrote user story cards. In this activity, each user writes on a small card one or more ideas for a software feature and how they would use it. A user story template often uses the following format: “As a <role>, I want <feature> so that <reason>.” Then, the group discusses each idea and uses color coding, etc., to identify how challenging or complicated a feature might be to develop. The coordinators organized the groups on the basis of similar roles and interests. Then, each group reported back to the full workshop, followed by a discussion to identify different types of users and to rank the ideas on the basis of difficulty and priority of need. For the highest priority ideas and user types, participants split into groups to do a second activity: user story mapping. In this part of the exercise, each group collaborated to draw on a large paper the story of a full user experience for their given software feature idea, imagining how their user type would navigate the software. These stories focused on imagined new features, needs for inputs, outputs, and data processing, as well as ideas for visualization and the user interface. Each group then presented their user story to the full workshop, which discussed and voted to decide which ideas would be prioritized for development (figure. 2).

conditions and eight future climate scenarios, close interaction with the development team was essential to undertake a fundamental reengineering of Wallace that enabled building separate SDMs for various species in the same session and performing some multispecies analyses (Kass et al. 2023). Frequent communication with the Wallace developers led to testing and bug reporting on the reengineered software and efficient identification, implementation, and testing of new downstream functionalities (e.g., using imported range estimates for diversity index calculations; Lemus-Mejía et al. 2021).

Finally, as a cross-cutting advance informed by the codesign, we also identified the need to connect the new Wallace directly with BioModelos (Velásquez-Tibatá et al. 2019), an existing key resource of biodiversity information for Colombia's decision-makers listed by the IUCN as a tool for red-listing (https://www.iucnredlist.org/resources/spatialtoolsanddata). For this integration, end users mapped out what data they wanted to pull from BioModelos to use in Wallace (expert-vetted species occurrence records) and what data and formats for SDMs and related calculations should be sent back to BioModelos. The respective development teams then made significant changes to the existing tools to meet these needs, including surmounting two new challenges: opening an external API for BioModelos and sending a customized payload from Wallace with standardized metadata that could be received by BioModelos. Serendipitously, because of its user-friendly visualization features, the expanded Wallace also made it easier and faster for Colombia Biodiversity Observation Network users to add vetted biodiversity information to BioModelos. These advances have enabled concrete real-world uses, because BioModelos products contribute to the *National Ecosystem Map of Colombia*, numerous volumes of the *Atlas of Colombian Biodiversity*, national assessments of species threats (as was mentioned above), and a monitoring information system for Colombia's National System of Protected Areas, which all inform national plans and policies developed by Colombia's National Council of Economic and Social Policy-CONPES (Velásquez-Tibatá et al. 2019, Chaves et al. 2020, Departamento Nacional de Planeación 2021).

## Lessons and recommendations

Broad inclusion in this process—and the funding and institutional backing to promote it—helped bridge the gap between the end users and the developers, facilitating the creation of usable needs-driven tools. This collaborative process represents a successful example of the flexible use of agile software development tailored to achieve broad representation from a given user community (Hohl et al. 2018), where familiarity and expertise with development frameworks and modeling methods varied greatly. Inclusion of more than 70 end user participants and beta testers, with a plurality of viewpoints and needs, slowed the design process by necessity but ultimately led to a highly useful product (figure 1). Although the codesign process takes longer, it can avoid problems such as overspecialized, inflexible tools (Siepel 2019) and a lack of uptake.

There is no singular framework to guide codesign approaches, which vary quite a bit on a spectrum from user-centered design to approaches that promote active user participation in codesign (Antonini 2021). In our experience, a highly participatory and cooperative approach was the most effective, and it was especially important to include mechanisms to make decision-making a cooperative process. For example, deciding priorities for development among different ideas for new software features and the resolution of any disagreements related to this were done as a large group through facilitated priority ranking activities and discussion (box 1) rather than via a top-down approach. It was also very important to our process that the idea for the collaboration was initiated from the very beginning via brainstorming between personnel at a Colombian institution, the Alexander von Humboldt Institute, and the software developers of Wallace, several of whom had interacted professionally in the past. Beginning this way set the stage for continuous, balanced, and effective collaboration throughout the codesign process.

Importantly, we found that only certain elements specified in typical agile software development methodologies (Hohl et al. 2018) were necessary in our case, especially those related to regular inclusion and communication with users (figure 1, steps B, D, and G). Indeed, we needed more flexibility in how and when the end users were engaged than in standard agile frameworks, with more frequent interactions than is typical. In contrast, following a formalized framework with concerted sprints for development of particular tasks (such as scrum; Hohl et al. 2018) was not necessary to achieve our goals. Such an approach would also have been extremely challenging, given that almost all members of the development team represented various academic or governmental groups and simultaneously worked on many other software, research, or administration tasks.

As we mentioned earlier, a key element of success was funding from institutions that recognized the need to support interactions among diverse end users and developers. In our case, private and public institutions—in particular, the Alexander von Humboldt Institute—provided extensive in-kind contributions (especially personnel time), and NASA and US National Science Foundation funding facilitated the formation of a multidisciplinary and intersectoral community focused on a common goal. This involved two in-person workshops (including travel from the United States and many parts of Colombia), a virtual workshop during the COVID-19 pandemic, and copious virtual small group meetings. Another key to success was the ability for developers and end users to communicate seamlessly. In our case, most developers and end users were fluent in both Spanish and English and could therefore interact with most others in their preferred language.

Once the expanded software was produced, training, user guidance, tutorials, and source code documentation constituted additional critical elements for the tool to reach a broad user community (Johnson et al. 2023, Kass et al. 2023, including a Spanish vignette). With governmental support, we could fund open-access disciplinary publications that document and explain the software packages behind the new functionalities (Merow et al. 2022, Galante et al. 2023) and provide training workshops, webinars, user support, and software maintenance (Merow et al. 2023). Toward longevity, making a tool freely available, open source, and modular promotes community-driven growth and uptake (Kass et al. 2023), as opposed to being dependent on proprietary tools (Siepel 2019).

In summary, a codesign approach mitigates common pitfalls in software development by bolstering the flexibility and scalability of the products. It promotes collaboration among many developers and users with complementary perspectives, leading to products that align with the specific needs of various stakeholders. In this way, software codevelopment, implementation, and maintenance aimed at bringing solutions to biodiversity and conservation issues can increase countries’ capacities to inform policy, meet 2030 targets and sustainable development goals (Gonzalez and Londoño 2022), and generally improve human well-being.

## Supplementary Material

biae097_Supplemental_File

## Author Biography

Mary E. Blair (mblair1@amnh.org), Peter Ersts, Daniel F. López-Lozano, and Bethany A. Johnson are affiliated with the Center for Biodiversity and Conservation at the American Museum of Natural History, in New York, New York, in the United States. Elkin A. Noguera-Urbano, Jose Manuel Ochoa-Quintero, Daniel F. López-Lozano, Hector M. Arango, Cristian A. Cruz-Rodríguez, María C. Londoño-Murcia, Carlos J. Muñoz-Rodríguez, María Helena Olaya-Rodríguez, Erika Suárez-Valencia, and Camilo A. Zapata-Martinez are affiliated with the Instituto de Investigación de Recursos Biológicos Alexander von Humboldt, in Bogotá, Distrito Capital, in Colombia. Andrea Paz is affiliated with the Department of Environmental Systems Science at the Institute of Integrative Biology, in Zürich, Switzerland, and, along with Cristian A. Cruz-Rodríguez, is also affiliated with the Département de Sciences Biologiques at the Université de Montréal, in Montréal, Québec, in Canada. Cristina Lopez-Gallego and Juan L. Parra are affiliated with the Instituto de Biología at the Universidad de Antioquia, in Medellín, Colombia. María Ángela Echeverry-Galvis and J. Nicolás Urbina-Cardona are affiliated with the Departamento de Ecología y Territorio in the Facultad de Estudios Ambientales y Rurales at Pontificia Universidad Javeriana, in Bogotá, Distrito Capital, in Colombia. Juan Zuloaga is affiliated with the Department of Biology at McGill University, in Montréal, Québec, in Canada. Pilar Rodríguez is affiliated with the Comisión Nacional para el Conocimiento y Uso de la Biodiversidad, in the Ciudad de México, México. Leonardo Lemus-Mejia is affiliated with ProCat Colombia, in Bogotá, Distrito Capital, in Colombia. Matthew E. Aiello-Lammens and Samuel Chang Triguero are affiliated with the Department of Environmental Studies and Science at Pace University, in Pleasantville, New York, in the United States. Leonardo Buitrago is affiliated with Latin America and Caribbean Regional Support, at the Global Biodiversity Information Facility and with the Universidad Nacional de Colombia, in Bogotá, Distrito Capital, in Colombia. Juan F. Díaz-Nieto and Valentina Grisales-Betancur are affiliated with the Natural Systems and Sustainability Area at the Universidad EAFIT, in Medellín, Colombia. Dairo Escobar is an independent researcher out of Bogotá, Distrito Capital, in Colombia. Valentina Grisales-Betancur is also affiliated with the El Globo Nature Reserve, Támesis, Colombia. Bethany A. Johnson, Gonzalo E. Pinilla-Buitrago, and Robert P. Anderson are affiliated with the Department of Biology at the City College of New York, at the City University of New York, in New York, New York, in the United States. Jamie M. Kass is affiliated with the Macroecology Laboratory in the Graduate School of Life Sciences at Tohoku University, in Sendai, Miyagi, Japan. Cory Merow is affiliated with the University of Connecticut, in Storrs, Connecticut, in the United States. Gonzalo E. Pinilla-Buitrago, and Robert P. Anderson are affiliated with the PhD program in Biology in the Graduate Center of the City University of New York, in New York, New York, in the United States. Nicolette S. Roach is affiliated with the Department of Ecology and Conservation Biology at Texas A&M University, in College Station, Texas, in the United States. Octavio Rojas-Soto is affiliated with the Instituto de Ecología AC, in Veracruz, México. Néstor Roncancio-Duque is affiliated with the Parque Nacional Natural Las Hermosas, in Palmira, and with the Instituto Amazónico de Investigaciones Científicas—Sinchi, in Inírida, Colombia. Jorge Velásquez-Tibatá is affiliated with Audubon Americas, at the National Audubon Society, in Bogotá, Distrito Capital, in Colombia. Robert P. Anderson is also affiliated with the Division of Vertebrate Zoology at the American Museum of Natural History, in New York, New York, in the United States.
