# Supplementary material for: Software codesign between end users and developers to enhance utility for biodiversity conservation
Source: Bioscience. 2024 Oct 22;74(12):867–73. doi: 10.1093/biosci/biae097 (PMC11660944; doi:10.1093/biosci/biae097)
Supplement: biae097_Supplemental_File [file biae097_supplemental_file.pdf]

Supplementary File 1. Spanish Translation / Traducción al español

Blair, M.E. et al. (2024). Software co-design between end-users and developers to enhance utility for biodiversity conservation. BioScience. DOI:10.1093/biosci/biae097

Título: ***Co-diseño de software entre usuarios finales y desarrolladores para potenciar su utilidad en la conservación de la biodiversidad***

Autores: Mary E. Blair[1*+], Elkin A. Noguera-Urbano[2+], José Manuel Ochoa-Quintero[2], Andrea Paz[3,4], Cristina Lopez-Gallego[5], María Ángela Echeverry-Galvis[6], Juan Zuloaga[7], Pilar Rodríguez[8], Leonardo Lemus-Mejia[9], Peter Ersts[1], Daniel F. López-Lozano[1,2], Matthew E. Aiello-Lammens[10], Héctor M. Arango[2], Leonardo Buitrago[11,12], Samuel Chang Triguero[10], Cristian A. Cruz-Rodríguez[2,4], Juan F. Díaz-Nieto[13], Dairo Escobar[14], Valentina Grisales-Betancur[13,15], Bethany A. Johnson[1,16], Jamie M. Kass[17], María C. Londoño-Murcia[2], Cory Merow[18], Carlos J. Muñoz-Rodríguez[2], María Helena Olaya-Rodríguez[2], Juan L. Parra[5], Gonzalo E. Pinilla-Buitrago[16,19], Nicolette S. Roach[20], Octavio Rojas-Soto[21], Néstor Roncancio-Duque[22,23], Erika Suárez-Valencia[2], J. Nicolás Urbina-Cardona[6], Jorge Velásquez-Tibatá[24], Camilo A. Zapata-Martinez[2], and Robert P. Anderson[16,19,25]

Afiliaciones:

[1]Center for Biodiversity and Conservation, American Museum of Natural History; New York, NY, USA

[2]Instituto de Investigación de Recursos Biológicos Alexander von Humboldt; Bogotá, D.C., Colombia

[3]Department of Environmental Systems Science, Institute of Integrative Biology, ETH Zürich; Zürich, Switzerland

[4]Département de Sciences Biologiques, Université de Montréal, Montréal, (QC), Canada
[5]Instituto de Biología, Universidad de Antioquia; Medellín, Colombia
[6]Departamento de Ecología y Territorio, Facultad de Estudios Ambientales y Rurales, Pontificia Universidad Javeriana; Bogotá, D.C., Colombia
[7]Department of Biology, McGill University; Montréal, (QC), Canada
[8]Comisión Nacional para el Conocimiento y Uso de la Biodiversidad (CONABIO); Ciudad de México, México
[9]ProCat Colombia; Bogotá, D.C., Colombia
[10]Department of Environmental Studies and Science, Pace University; Pleasantville, NY, USA
[11]Latin America and Caribbean Regional Support, The Global Biodiversity Information Facility; Bogotá, D.C., Colombia
[12]Universidad Nacional de Colombia; Bogotá, D.C., Colombia
[13]Natural Systems and Sustainability Area, Universidad EAFIT; Medellín, Colombia
[14]Independent Researcher, Bogotá, D.C., Colombia
[15]El Globo Nature Reserve, Támesis, Colombia
[16]Department of Biology, City College of New York, City University of New York, New York, NY, USA
[17]Macroecology Laboratory, Graduate School of Life Sciences, Tohoku University, Sendai, Miyagi, Japan
[18]University of Connecticut; Storrs, CT, USA

[19]Ph.D. Program in Biology, Graduate Center, City University of New York, New York, NY, USA

[20]Department of Ecology and Conservation Biology, Texas A&M University, College Station, TX, USA

[21]Instituto de Ecología A.C. (INECOL); Veracruz, México

[22]Parque Nacional Natural Las Hermosas, Palmira, Colombia

[23]Instituto Amazónico de Investigaciones Científicas – Sinchi, Inírida, Colombia

[24]Audubon Americas, National Audubon Society; Bogotá, D.C., Colombia

[25]Division of Vertebrate Zoology, American Museum of Natural History, New York, NY, USA

[+]Estos autores contribuyeron igualitariamente al manuscrito.

*Autor de correspondencia. Correo: mblair1@amnh.org

Palabras clave: ecología aplicada, biodiversidad, biogeografía, conservación, informática

Resumen

La creación de herramientas de software que respondan a las necesidades de una amplia gama de tomadores de decisiones requiere la inclusión de diferentes perspectivas a lo largo del proceso de desarrollo. Las herramientas de software para la conservación de la biodiversidad a menudo no cumplen con este objetivo, en parte porque las necesidades de los tomadores de decisiones pueden superar las capacidades de los equipos de investigación o incluso de las instituciones. Aquí mostramos que el co-diseño participativo y colaborativo optimiza la utilidad de las herramientas de software para una mejor toma de decisiones en la planificación de la conservación de la biodiversidad, como lo demuestran nuestras experiencias en el desarrollo

de un conjunto de herramientas integradas para el caso de Colombia. En concreto, colaboramos interdisciplinaria e interinstitucionalmente entre modeladores ecológicos, ingenieros de software y un perfil diverso de usuarios finales potenciales, incluidos tomadores de decisiones, practicantes de conservación y expertos en biodiversidad. Aprovechamos y modificamos paradigmas comunes de producción de software, incluido el co-diseño y el desarrollo ágil, para facilitar la colaboración en todas las etapas (incluyendo la conceptualización, el desarrollo, las pruebas y la retroalimentación) y asegurar la accesibilidad y aplicabilidad de las nuevas herramientas para soportar la toma de decisiones informada en la planificación de la conservación de la biodiversidad.

Introducción

Las respuestas ágiles de política para conservar la biodiversidad, como cumplir con los objetivos del Marco Mundial de Biodiversidad para 2030, solo son posibles con información proveniente de marcos analíticos adaptables que enlacen múltiples fuentes de datos (González y Londoño, 2022). Esta información es esencial para las evaluaciones globales de biodiversidad, como las lideradas por IPBES (Plataforma Intergubernamental de Ciencia-Política sobre Biodiversidad y Servicios Ecosistémicos) y la UICN (Unión Internacional para la Conservación de la Naturaleza), y también sirve como insumo para informar a los tomadores de decisiones a diferentes escalas. Con este fin, las redes de observación y recopilación de datos de biodiversidad (González et al., 2023) obtienen, canalizan y facilitan el análisis casi en tiempo real de datos derivados del campo y observaciones mediante sensores remotos (SR) para cuantificar el estado de la biodiversidad y predecir escenarios para su futuro.

En general, la creación de herramientas de software que aborden las necesidades de una amplia gama de tomadores de decisiones requiere la inclusión de diferentes perspectivas a lo largo del proceso de desarrollo. Las herramientas de software para la conservación de la biodiversidad a menudo fallan en este sentido, en parte, porque las necesidades de los

tomadores de decisiones pueden superar las capacidades de los conjuntos de herramientas de grupos de investigación individuales o incluso de instituciones. Aquí mostramos que el co-diseño colaborativo y participativo entre muchas instituciones mejora la utilidad de las herramientas de software para una mejor toma de decisiones en la planificación de la conservación de la biodiversidad, como lo muestran nuestras experiencias en el desarrollo de un conjunto de herramientas integradas en Colombia. Específicamente, somos un equipo de modeladores ecológicos, ingenieros de software, expertos en biodiversidad y practicantes de conservación de una amplia gama de disciplinas, antecedentes e instituciones que se unieron para satisfacer las necesidades de la Red de Observación de Biodiversidad de Colombia (https://geobon.org/bons/national-regional-bon/national-bon/colombia-bon/). Los autores de este trabajo incluyen tanto a usuarios finales como a desarrolladores con una amplia gama de habilidades y conocimientos, reunidos con el objetivo de aprovechar y modificar paradigmas comunes de producción de software, incluidos el co-diseño y el desarrollo ágil (Hohl et al., 2018). Durante este proceso, facilitamos la colaboración e inclusión de los autores a lo largo de todas las etapas de nuestros esfuerzos (incluyendo la conceptualización, desarrollo, pruebas y retroalimentación) para asegurar la accesibilidad, aplicabilidad e impacto de las nuevas herramientas.

Co-diseño de software para la planificación de la conservación de la biodiversidad

Nuestras experiencias derivan de actividades bajo la Red de Observación de Biodiversidad del Grupo de Observaciones de la Tierra de (GEO BON). GEO BON facilita el desarrollo de Redes de Observación de Biodiversidad (BONs) nacionales, regionales y temáticos (González et al., 2023) y herramientas de software relacionadas para conectar a los usuarios finales y desarrolladores de herramientas de software en todo el mundo. Dentro de GEO BON se estandarizó una serie de Variables Esenciales de la Biodiversidad (VEBs) para armonizar el monitoreo aprovechando los conjuntos de datos de SR (Pereira et al., 2013). Iniciativas de

financiación estratégica para conectar las VEBs con la toma de decisiones, como el programa de la NASA que financió nuestros esfuerzos, han promovido el desarrollo de sistemas de apoyo a la toma de decisiones: por medio de la generación de herramientas de software que tienen como objetivo rastrear las VEBs para respaldar aplicaciones específicas en la toma de decisiones (GEO BON, 2017).

Los modelos de distribución de especies (MDEs) estiman la idoneidad ambiental y son útiles para estimar la VEB de Distribución de Especies (Pereira et al., 2013; Vihervaara et al., 2017; Urbina-Cardona et al., 2019). Los MDEs aprovechan los datos de biodiversidad (registros de ocurrencia de una especie) y pueden guiar la toma de decisiones en conservación, especialmente cuando integran el conocimiento de expertos (Araújo et al., 2019; Merow et al., 2022; Velásquez-Tibatá et al., 2019). El procesamiento posterior de los MDEs puede aumentar su utilidad para la toma de decisiones en conservación de la biodiversidad al incorporar información actual de sensores remotos y otros factores que reflejan el uso del suelo, la sobreexplotación u otros factores adicionales (Merow et al., 2022). También pueden extrapolarse en el espacio y el tiempo para pronosticar invasiones o cambios en la biodiversidad bajo escenarios climáticos y de uso del suelo dinámicos. Sin embargo, hasta hace poco, construir MDEs de alta calidad implicaba una curva de aprendizaje pronunciada y su implementación en sistemas de apoyo en campo había sido limitada por la falta de herramientas de software didácticas y fáciles de usar, así como por la falta de marcos para la curación de registros de entrada, y la construcción y procesamiento posterior de los MDEs (González y Londoño, 2022).

Aquí aprovechamos dos herramientas innovadoras y reunimos a un conjunto amplio de usuarios finales y desarrolladores para co-crear un software que cumpla con las necesidades de cuantificar la VEB de Distribución de Especies. De manera crítica, el BON Colombia (González et al., 2023) construye y mantiene una comunidad activa para desarrollar Sistemas de Información sobre Biodiversidad a escalas nacionales y subnacionales. Con el fin de

promover las evaluaciones de indicadores de cambio en la biodiversidad, nos asociamos con el BON Colombia para mejorar la aplicación de construcción de MDEs *Wallace EcoMod*, caracterizada por ser de código abierto, fácil de usar y modular (Kass et al., 2018, 2023; en adelante, *Wallace*). Juntos identificamos la necesidad de lograr la interoperabilidad entre *Wallace* y *BioModelos*, la aplicación existente en el BON Colombia para la curaduría por parte de expertos de conjuntos de datos de ocurrencias de especies y el modelamiento de sus distribuciones (Velásquez-Tibatá et al., 2019). Sinérgicamente, estas conexiones permitirían a los usuarios cargar datos de ocurrencia validados desde *BioModelos* en *Wallace*, y enviar estimaciones de rangos de MDEs procesados posteriormente en *Wallace* de vuelta a *BioModelos*. Para lograr esto, reunimos a un grupo diverso para co-diseñar y desarrollar paquetes de software que aprovechen los datos de SR para procesar los resultados de los MDEs con el objetivo de estimar los rangos de distribución actuales de las especies y calcular indicadores de cambio en la biodiversidad: *maskRangeR* (Merow et al., 2022) y *changeRangeR* (Galante et al., 2023). Luego integramos estos paquetes como nuevos módulos en *Wallace*, permitiendo informes sobre el estado, las tendencias y los impulsores de cambio en la biodiversidad, realizados a partir de análisis accesibles, documentados y reproducibles (https://github.com/wallaceEcoMod/wallace/tree/biomodelos). Además, habilitamos la publicación de datos (push) y la recuperación (pull) desde y hacia la API de *BioModelos*.

Durante el proceso iterativo de co-diseño, desarrollo y pruebas, los desarrolladores de software se involucraron con los usuarios finales (Fig. 1). Combinando aspectos de los paradigmas de co-diseño y desarrollo ágil, este proceso incluyó ciclos de actividades de participación en las que los usuarios comunicaron sus necesidades y objetivos de forma explícita; por ejemplo, mapeo de usuarios y visualización, lanzamientos y pruebas de versiones alfa y beta e integración de actualizaciones ágiles (Brown, 2008; Hohl et al., 2018). Para mejorar el potencial de accesibilidad, aplicabilidad e impacto amplio de la nuevas herramientas, el equipo incluyó a usuarios finales de una amplia variedad de sectores y organizaciones (e.g.,

ONGs, organizaciones gubernamentales, academia) con diferentes tipos de decisiones de manejo que querían informar (e.g., planificación de áreas protegidas, monitoreo, compensaciones de impacto ambiental).

[Fig. 1]

Estudios de caso

Como primer ejemplo de un contexto de usuario que informó el co-diseño (Fig. 1; Paso A), nuestro equipo incluyó practicantes responsables de evaluaciones nacionales y globales del riesgo de extinción de especies (e.g., listas rojas globales y nacionales). Las evaluaciones de riesgo de extinción, como las de la UICN, idealmente se realizan con información sobre el tamaño de la población, la distribución geográfica y las tendencias temporales asociadas (UICN, 2024). Sin embargo, en la mayoría de los casos, solo se dispone de información aproximada sobre las distribuciones actuales de las especies (Anderson, 2023). En contraste, los MDEs pueden proporcionar información tanto de las distribuciones actuales como potencialmente futuras de las especies, y los productos de SR pueden aprovecharse en el procesamiento posterior para cuantificar las tendencias temporales, como la pérdida reciente de hábitat y el riesgo de extinción. Aunque algunos paquetes de software existentes proporcionan herramientas relevantes para calcular métricas espaciales, como el área de ocupación de la UICN (e.g., *conR*), los análisis de los impactos de amenazas particulares suelen ser solo cualitativos. El grupo observó que tales análisis podrían cuantificarse y compararse en el tiempo utilizando productos de SR si se creara una herramienta accesible para los practicantes.

A través de ejercicios de visualización de usuarios (Fig. 2, Cuadro 1), exploramos opciones para mejorar *Wallace* de manera que pudiera aprovechar los MDEs para contribuir a las evaluaciones de riesgo de extinción y a los planes de conservación (Fig. 1, Paso B).

Diseñamos un flujo de trabajo de procesamiento posterior para combinar los MDEs con información sobre la cantidad y calidad del hábitat a partir de capas derivadas de SR como la huella humana (Correa-Ayram, et al., 2020), para informar las evaluaciones de listas rojas y ponderar los impactos potenciales de amenazas específicas (como la degradación del hábitat) y las acciones de conservación (como la cobertura en áreas protegidas). A partir de esta visión, desarrollamos los paquetes R *maskRangeR* y *changeRangeR* y los agregamos a *Wallace* (Fig. 1, Paso C). Los paquetes y su inclusión en *Wallace* fueron probados con varias rondas de retroalimentación y ajustes (Fig. 1, Pasos D y E) para estimar métricas de listas rojas utilizando MDEs y cuantificar algunos aspectos de la incertidumbre. En total, después del taller presencial inicial para la visualización, convocamos dos adicionales (uno presencial y otro virtual), centrados principalmente en probar nuevas características y recibir retroalimentación de los usuarios para realizar más mejoras. El equipo de desarrollo también distribuyó un formulario en línea para pruebas y retroalimentación con el fin de llegar a un grupo más amplio de usuarios, además de realizar al menos diez reuniones adicionales más pequeñas, con usuarios específicos, para obtener retroalimentación detallada sobre características particulares, necesidades e inconvenientes relacionados con la implementación y los casos de uso.

En última instancia, los nuevos paquetes y su integración en *Wallace* permitieron a los usuarios estandarizar sus análisis sobre los efectos de las amenazas y las acciones de conservación en las distribuciones de especies al proporcionar información cuantitativa y espacialmente explícita. Esto resultó en información clave para las evaluaciones que apoyaron la Lista Nacional de Especies Silvestres Amenazadas de Colombia (MADS, 2024), que incluye evaluaciones para plantas (López-Gallego y Morales-Morales, 2023), primates (Henao-Díaz et al., 2020), roedores (Ramírez-Chaves et al., 2022) y aves (Chaparro-Herrera et al., 2024), apoyando la asignación adecuada de responsabilidades de conservación a las autoridades locales, con base en información espacialmente explícita y validada por expertos sobre las distribuciones de especies amenazadas.

[Fig. 2]

[Inicio de Cuadro 1]

Cuadro 1. Ejercicios de visualización de usuarios. Los ejercicios de visualización de usuarios, al igual que el mapeo de historias (*story mapping*), se utilizan a menudo en enfoques de diseño de software centrado en el usuario, como el desarrollo ágil de software. Adoptamos un enfoque altamente participativo para implementar estos ejercicios. En un taller de consulta de usuarios de varios días, los coordinadores involucraron a practicantes de conservación y expertos en biodiversidad en dos ejercicios planteados para generar, presentar y priorizar visiones para el software en desarrollo, considerando sus necesidades. Primero, en pequeños grupos, los participantes escribieron "tarjetas de historias de usuario". En esta actividad, cada usuario escribe en una tarjeta pequeña una o más ideas para una funcionalidad de software y cómo la utilizaría. Un formato típico de historia de usuario es el siguiente: "Como <rol>, quiero <funcionalidad> para que <razón>". Luego, el grupo discute cada idea y utiliza codificación de colores, etc., para identificar qué tan desafiante o complicada podría ser el desarrollo de una función. Los coordinadores organizaron grupos basados en roles e intereses similares. Luego, cada grupo presentó los resultados al taller completo, seguido de una discusión para identificar diferentes tipos de usuarios y clasificar las ideas según la dificultad y prioridad de necesidad. Para las ideas y tipos de usuarios con mayor prioridad, los participantes se dividieron en grupos para realizar una segunda actividad: mapeo de historias de usuario. Aquí, cada grupo colaboró para dibujar en un gran papel la historia de una experiencia de usuario completa para la funcionalidad de software que se les asignó, imaginando cómo su "tipo de usuario" navegaría por el software. Estas historias se centraron en nuevas funciones imaginadas, necesidades de entradas, salidas y procesamiento de datos, así como ideas para la visualización y la interfaz

de usuario. Luego, cada grupo presentó su historia de usuario al taller completo, que discutió y votó para decidir qué ideas se priorizarían para el desarrollo [Fig. 2].

[Fin Cuadro 1]

Mientras que el contexto de la lista roja informó el ciclo inicial de desarrollo, nuestro grupo diverso también incluyó usuarios cuyas necesidades guiaron mejoras adicionales para un uso más extenso y la aplicabilidad a una gama más amplia de contextos de toma de decisiones en conservación. Esto se facilitó desarrollando estudios de caso adicionales (casos de uso; Fig. 1) realizando múltiples iteraciones de todo el ciclo después de ampliar la participación de la comunidad (Fig. 1, Paso H). En un contexto de usuario adicional que informó el co-diseño, los investigadores quisieron estimar y mapear la riqueza de especies, el endemismo y la diversidad funcional para mejorar la selección de estrategias de planificación de la conservación bajo el cambio climático. Habían explorado otras herramientas para este propósito, pero detuvieron sus esfuerzos debido a las curvas de aprendizaje pronunciadas y las demandas computacionales. Con el objetivo específico de comparar las distribuciones potenciales de 135 especies de mamíferos bajo condiciones actuales y ocho escenarios climáticos futuros, la interacción cercana con el equipo de desarrollo fue esencial para emprender una reingeniería fundamental de *Wallace* que permitiera construir MDEs separados para varias especies en la misma sesión y realizar algunos análisis de múltiples especies (Kass et al., 2023). La comunicación frecuente con los desarrolladores de *Wallace* permitió realizar pruebas e informes de errores sobre el rediseño del software y la identificación eficiente, implementación y testeo de nuevas funcionalidades emergentes (por ejemplo, utilizando estimaciones de rango importadas para cálculos de índices de diversidad; Lemus-Mejía et al., 2021).

Finalmente, como un avance transversal informado por el co-diseño, también identificamos la necesidad de conectar el nuevo *Wallace* directamente con *BioModelos* (Velásquez-Tibatá et al., 2019), un recurso clave existente de información sobre biodiversidad para los tomadores de

decisiones de Colombia que está reconocido por la UICN como una herramienta para la evaluación de riesgo de extinción (https://www.iucnredlist.org/resources/spatialtoolsanddata). Para esta integración, los usuarios finales mapearon qué datos querían extraer de *BioModelos* para usar en *Wallace* (registros de ocurrencia de especies curados por expertos) y qué datos y formatos para los MDEs y cálculos relacionados deberían enviarse de vuelta a *BioModelos*. Los respectivos equipos de desarrollo hicieron luego cambios significativos en las herramientas existentes para satisfacer estas necesidades, incluyendo superar dos nuevos desafíos: 1) abrir una interfaz de programación de aplicaciones (API) externa para *BioModelos*, y 2) enviar una carga personalizada desde *Wallace* con metadatos estandarizados que pudieran ser recibidos por *BioModelos*. De manera fortuita, debido a sus características de visualización amigables para el usuario, la nueva versión expandida de *Wallace* también facilitó y aceleró que los usuarios del BON Colombia añadieran información examinada sobre biodiversidad a *BioModelos*. Estos avances han permitido usos concretos en el mundo real, ya que los productos de *BioModelos* contribuyen al Mapa Nacional de Ecosistemas de Colombia, numerosos volúmenes del Atlas de la Biodiversidad Colombiana, evaluaciones nacionales de especies amenazadas (como se mencionó anteriormente) y un Sistema de Información de Monitoreo para el Sistema de Áreas Protegidas de Colombia, los cuales, en conjunto, apoyan la formulación de los planes nacionales y políticas desarrolladas por el Consejo Nacional de Política Económica Social de Colombia -CONPES (Velásquez-Tibatá et al., 2019; Chaves et al., 2020; Departamento Nacional de Planeación, 2021).

Lecciones y recomendaciones

La inclusión amplia en este proceso, junto con el respaldo financiero e institucional para promoverlo, ayudó a cerrar la brecha entre los usuarios finales y los desarrolladores, facilitando la creación de herramientas usables y orientadas a responder necesidades. Este proceso colaborativo representa un ejemplo exitoso del uso flexible del desarrollo ágil de software

adaptado para lograr una amplia representación de una comunidad de usuarios dada (Hohl et al., 2018), donde la familiaridad y experiencia con los marcos de desarrollo y los métodos de modelado variaba considerablemente. La inclusión de más de 70 participantes: usuarios finales y probadores beta, con una heterogeneidad de puntos de vista y necesidades ralentizó el proceso de diseño por necesidad, pero en última instancia condujo a un producto altamente útil (Fig. 1). Aunque el proceso de co-diseño lleva más tiempo, puede evitar problemas como generación de herramientas sobre-especializadas, inflexibles y su falta de adopción.

No existe un marco único para guiar los enfoques de co-diseño, los cuales varían bastante en un espectro, desde el diseño centrado en el usuario hasta enfoques que promueven la participación activa de los usuarios en el co-diseño (Antonini, 2021). En nuestra experiencia, un enfoque altamente participativo y cooperativo fue el más eficaz, y fue especialmente importante incluir mecanismos para hacer de la toma de decisiones un proceso cooperativo. Por ejemplo, la decisión de prioridades para el desarrollo entre diferentes ideas para nuevas características de software, junto con la resolución de cualquier desacuerdo relacionado con esto, se implementaron en un grupo grande a través de actividades de priorización facilitada y discusión (Cuadro 1), en lugar de usar un enfoque de arriba hacia abajo. También fue muy importante para nuestro proceso que la idea de la colaboración se iniciara desde el principio a través de sesiones de lluvia de ideas entre el personal de una institución colombiana, el Instituto Alexander von Humboldt, y los desarrolladores de software de *Wallace*, varios de los cuales habían interactuado profesionalmente en el pasado. Comenzar de esta manera sentó las bases para una colaboración continua, equilibrada y eficaz durante todo el proceso de co-diseño.

Es importante destacar que solo algunos elementos especificados en las metodologías típicas de desarrollo ágil de software (Hohl et al., 2018) fueron necesarios en nuestro caso, especialmente aquellos relacionados con la inclusión y comunicación regular con los usuarios (Fig. 1, Pasos B, D, G). De hecho, necesitamos más flexibilidad en cómo y cuándo se involucró a los usuarios finales que en los marcos ágiles estándar, con interacciones más frecuentes de

lo habitual. En contraste, seguir un marco formalizado con iteraciones (*sprints*) concertados para el desarrollo de tareas particulares (como Scrum; Hohl et al., 2018) no fue necesario para lograr nuestros objetivos. Tal enfoque también habría sido extremadamente desafiante dado que casi todos los miembros del equipo de desarrollo representaban varios grupos académicos o gubernamentales y trabajaban simultáneamente en otras tareas de software, investigación o administración.

Como se mencionó, un elemento clave del éxito fue la financiación de instituciones que reconocieron la necesidad de apoyar las interacciones entre diversos usuarios finales y desarrolladores. En nuestro caso, instituciones privadas y públicas, en particular el Instituto Alexander von Humboldt, proporcionaron extensas contribuciones en especie (especialmente tiempo del personal) y la financiación de la NASA y la NSF facilitó la formación de una comunidad multidisciplinaria e intersectorial centrada en un objetivo común. Esto implicó dos talleres presenciales (incluido el viaje desde los EE.UU. y muchas partes de Colombia), un taller virtual durante la pandemia de COVID-19 y numerosas reuniones virtuales en grupos pequeños. Otro factor clave para el éxito fue la capacidad de los desarrolladores y usuarios finales para comunicarse sin problemas. En nuestro caso, la mayoría de los desarrolladores y usuarios finales eran fluidos en español e inglés, lo que les permitió interactuar con los demás en su idioma preferido.

Con la producción del software expandido, se generaron elementos adicionales críticos para que la herramienta llegara a una amplia comunidad de usuarios, como el caso de capacitaciones, las guías para usuarios, tutoriales y la documentación del código fuente (Johnson et al., 2023; Kass et al., 2023, incluida una viñeta en español). Con el apoyo gubernamental, pudimos financiar publicaciones disciplinarias de acceso abierto que documentan y explican los paquetes de software detrás de las nuevas funcionalidades (Merow et al., 2022; Galante et al., 2023) y proporcionar talleres de capacitación, seminarios web, soporte para usuarios y mantenimiento del software (Merow et al., 2023). Pensando en el largo

plazo, hacer que una herramienta esté disponible gratuitamente, en código abierto y modular, promueve el crecimiento impulsado por la comunidad y su adopción (Kass et al., 2023), en lugar de depender de herramientas propietarias (Siepel, 2019).

En resumen, un enfoque de co-diseño mitiga los errores comunes en el desarrollo de software al fortalecer la flexibilidad y escalabilidad de los productos. Promueve la colaboración entre muchos desarrolladores y usuarios con perspectivas complementarias, lo que conduce a productos que se alinean con las necesidades específicas de varios grupos de interés. De esta manera, el co-desarrollo, implementación y mantenimiento de software orientado a brindar soluciones a los problemas de biodiversidad y conservación puede aumentar la capacidad de los países para informar políticas, cumplir con los objetivos de 2030 y los objetivos de desarrollo sostenible (González y Londoño, 2022) y, en general, mejorar el bienestar humano.

Referencias

Anderson RP. 2023. Integrating habitat-masked range maps with quantifications of prevalence to estimate area of occupancy in IUCN assessments. Conservation Biology, 37, e14019. doi:10.1111/cobi.14019

Antonini M. 2021. An overview of co-design: Advantages, challenges and perspectives of users' involvement in the design process. Journal of Design Thinking 2(1): 45-60.

Araújo MB, Anderson RP, Barbosa AM, et al. 2019. Standards for distribution models in biodiversity assessments. Science Advances, 5(1): eaat4858. doi:10.1126/sciadv.aat4858

Brown T. 2008. Design thinking. Harvard Business Review, 86(6): 84-94.

Chaves ME, Santamaría M, et al. 2020. Conceptos y herramientas para transitar hacia la sostenibilidad. Avances a 2020. Instituto de Investigación de Recursos Biológicos Alexander von Humboldt. Bogotá D.C. Colombia.

Chaparro-Herrera S, Acevedo-Charry O, Ocampo D, Echeverry-Galvis M. et al. 2024. Atlas de la biodiversidad de Colombia. Aves Endémicas. Instituto de Investigación de Recursos Biológicos Alexander von Humboldt. Bogotá D. C., Colombia. 100 pp.

Correa Ayram, C. A., Etter, A., Díaz-Timoté, J., Rodríguez Buriticá, S., Ramírez, W., & Corzo, G. (2020). Spatiotemporal evaluation of the human footprint in Colombia: Four decades of anthropic impact in highly biodiverse ecosystems. *Ecological Indicators*, *117*, 106630. https://doi.org/https://doi.org/10.1016/j.ecolind.2020.106630.

Departamento Nacional de Planeación. 2021. Política para la Consolidación del Sistema Nacional de Áreas Protegidas- SINAP (Documento CONPES 4050). Bogotá D.C. Colombia DNP. (2021 September 27)

Galante PJ, Chang S, Paz A, et al. 2023. changeRangeR: an R package for reproducible biodiversity change metrics from species distribution estimates. Conservation Science & Practice 5(1): e12863. doi:10.1111/csp2.12863

GEO BON Secretariat. 2017. NASA announces new funding for GEO BON activities. https://geobon.org/nasa-announces-new-funding-for-geo-bon-activities/ (Accessed 29 Sep 2023).

Gonzalez A, Londoño MC. 2022. Monitor biodiversity for action. Science 378(6625): 1147. doi:10.1126/science.adg1506

Gonzalez A, Vihervaara P, Balvanera P, et al. 2023. A global biodiversity observing system to unite monitoring and guide action. Nature Ecology & Evolution. https://doi.org/10.1038/s41559-023-02171-0

Henao-Díaz F, et al. (2020). Atlas de la biodiversidad de Colombia. Primates. Instituto de Investigación de Recursos Biológicos Alexander von Humboldt. Bogotá D. C., Colombia. 51 pp.

Hohl P, Klünder J, van Bennekum A, et al. 2018. Back to the future: origins and directions of the "Agile Manifesto" – views of the originators. Journal of Software Engineering Research and Development 6: 15. doi:10.1186/s40411-018-0059-z

IUCN. 2024. Guidelines for using the IUCN Red List categories and criteria, version 16. https://www.iucnredlist.org/documents/RedListGuidelines.pdf

Johnson BA, Pinilla-Buitrago GE, Paz A, Kass JM, Meenan SI, and Anderson RP. 2023. Creating and Optimizing Species Distribution Models: A Vignette for Wallace Ecological Modeling Application v2.0. Lessons in Conservation 13(1): 76-118. https://ncep.amnh.org/linc Full teaching module available at: https://doi.org/10.5531/cbc.ncep.0184 En español: https://doi.org/10.5531/cbc.ncep.0185

Kass JM, Vilela B, Aiello-Lammens ME, et al. 2018. Wallace: A flexible platform for reproducible modeling of species niches and distributions built for community expansion. Methods in Ecology and Evolution, 9, 1151–1156.

Kass JM, Pinilla-Buitrago G, Paz A, et al. 2023. Wallace 2: a shiny app for modeling species niches and distributions redesigned to facilitate expansion via module contributions. Ecography 2023(3): e06547. doi:10.1111/ecog.06547

Lemus-Mejía L, Paredes-Casas C, Gómez-Junco G, et al. 2021. Distribución potencial de mamíferos: un análisis ante escenarios de Cambio climático en Cundinamarca, Colombia. P. 162 In Lizcano DL, Concha-Osbahr DC, Ramírez-Chaves HE, et al. IV Congreso Colombiano de Mastozoología - Libro de Resúmenes. Sociedad Colombiana de Mastozoología (SCMas). Bogotá, Colombia. doi.org10.47603/mano.v7n3.312

Lopez-Gallego C, Morales-Morales PA. 2023. The Red List for the endemic trees of Colombia: Effective conservation targeted for plants required in biodiversity hotspots. Plants, People, Planet, 5(4): 617–627. doi.org:10.1002/ppp3.10360

Merow C, Galante PJ, Kass JM, et al. 2022. Operationalizing expert knowledge in species' range estimates using diverse data types. Frontiers of Biogeography 14.2: e53589.

Merow C, Boyle B, Enquist BM, et al. 2023. Better incentives are needed to reward academic software development. Nature Ecology & Evolution 7: 626-627. doi: 10.1038/s41559-023-02008-w

Ministerio de Ambiente y Desarrollo Sostenible (MADS). 2024. Lista de especies silvestres amenazadas de la diversidad biológica continental y marino-costera de Colombia - Resolución 0126 de 2024 expedida por el Ministerio de Ambiente y Desarrollo Sostenible. v1.0. Ministerio de Ambiente y Desarrollo Sostenible - MADS. Dataset/Checklist. https://doi.org/10.15472/frowz3

Pereira HM, Ferrier S, Walters M, et al. 2013. Essential Biodiversity Variables. Science 339(6117): 277-278. doi: 10.1126/science.1229931

Ramírez-Chaves HE, et al. 2022. Atlas de la biodiversidad de Colombia. Grandes Roedores. Instituto de Investigación de Recursos Biológicos Alexander von Humboldt. Bogotá D. C., Colombia. 31 pp.

Siepel A. 2019. Challenges in funding and developing genomic software: roots and remedies. Genome Biology 20: 147. doi:10.1186/s13059-019-1763-7

Urbina-Cardona N, Londoño MC, Blair ME, Velásquez J, Loyola R, and Morales-Devia H. 2019. Species distribution modeling in Latin America: a 25-year retrospective review. Tropical Conservation Science 12: 1-9. doi: 10.1177/1940082919854058

Vihervaara P, Auvinen AP, Mononen L, et al. 2017. How essential biodiversity variables and remote sensing can help national biodiversity monitoring. Global Ecology and Conservation 10: 43-59.

Velásquez-Tibatá J, Olaya-Rodríguez MH, López-Lozano D, et al. 2019. BioModelos: A collaborative online system to map species distributions. PLoS ONE, 14(3): e0214522. doi:10.1371/journal.pone.0214522

Agradecimientos: Agradecemos a Peter J. Galante, Nadav Gazit, Beth E. Gerstner, Ned Horning y Erica E. Johnson por ayudar a dar forma a estas ideas y a Cynthia L. Schmidt por proporcionar comentarios sobre este manuscrito. También estamos agradecidos por los comentarios proporcionados por otros participantes de nuestros talleres de consulta y pruebas con usuarios finales, especialmente Henry Agudelo, William J. Agudelo, Silvia Álvarez, Daniel Amariles, María F. Batista Morales, Carolina Castellanos, Lina Estupiñán, Iván González, José González-Maya, Victor Gutiérrez-Vélez, Patrick Jantz, Edgardo Londoño-Cruz, Silvia López Casas, Paula Morales, Julián Ramírez, Susana Rodríguez Buriticá, Nelson Salinas, y todas las instituciones de nuestros participantes: Asociación Colombiana de Herpetología, Asociación Colombiana de Ornitología, Asociación Primatológica Colombiana, Comisión Nacional para el Conocimiento y Uso de la Biodiversidad-México, Instituto de Ecología A.C., McGill University, National Audubon Society, Parques Nacionales Naturales de Colombia, Sistema de Información sobre Biodiversidad de Colombia, Sociedad Colombiana de Mastozoología, Temple University, Texas A&M University, The Nature Conservancy—Colombia, Universidad Nacional de Colombia, Pontificia Universidad Javeriana, Universidad de Antioquia, Universidad del Valle, Universidad EAFIT, y Wildlife Conservation Society-Colombia, entre otras. JZ quisiera agradecer a la Liber Ero Chair en Conservación de la Biodiversidad en Canadá por su apoyo.

Financiación: Esta investigación fue financiada por la National Aeronautics and Space Administración, con los proyectos 80NSSC18K0406 y 80NSSC21K1351 (MEB), y la National Science Foundation, con el proyecto DBI-1661510 (RPA).

Contribución de los autores:

Conceptualización: AP, CLG, CM, EAN-U, GEPB, JMK, JMO-Q, JV-T, MAE, MEA-L, MEB, MCL-M, RPA.

Metodología: AP, CLG, CM, DE, EAN-U, GEPB, HMA, JFD-N, JLP, LBMEA-L, MEB,

MCL-M, MHO-R, NSR, NR-D, NU-C, OR-S, PE, RPA.

Software: AP, BAJ, CAC-R, CAZ-M, CJM-R, CM, DFL-L, EAN-U, ES-V, GEPB, MEA-L, MEB, JMK, PE, RPA, SCT.

Investigación: BAJ, CLG, MAE, LL, VG-B.

Validación: BAJ, CLG, HMA, JFD-N, JZ, LL, MAE, NSR, NR-D, NU-C, PR.

Visualización: EAN-U, JZ, MEB.

Adquisición de financiación: CM, JV-T, MEA-L, MEB, RPA.

Administración del proyecto: EAN-U, JMO, JV, MEB, RPA.

Redacción – borrador original: AP, CLG, EAN-U, JMO-Q, JZ, LL, MAE, MEB, PR, RPA.

Redacción – revisión y edición: todos los autores.

Conflictos de intereses: Los autores declaran no tener conflictos de intereses.

Disponibilidad de datos y materiales: Todo el código está disponible en: https://github.com/wallaceEcoMod/wallace/tree/biomodelos y también a través de: https://boninabox.geobon.org/tools

Figuras.

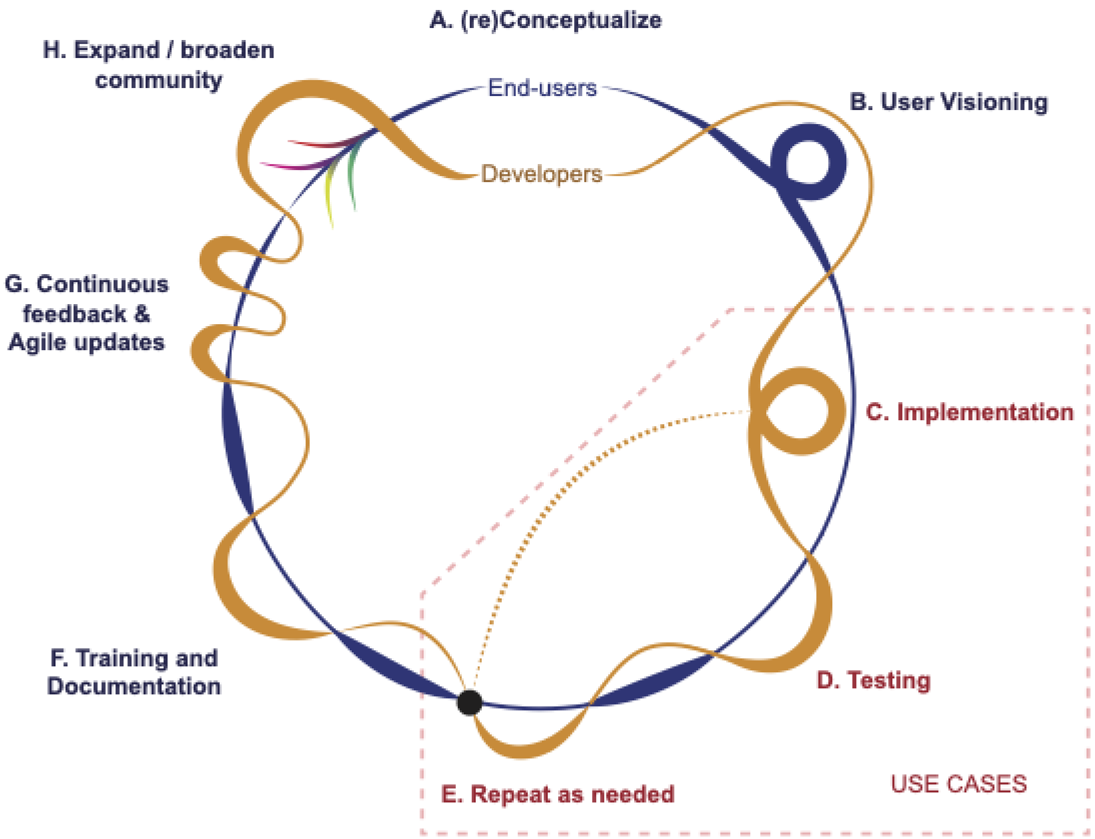

Fig. 1. Fases en nuestra implementación flexible de los pasos centrales del desarrollo ágil de software para lograr el co-diseño de extensiones de *Wallace EcoMod* y *BioModelos* para usuarios enfocados en conservación de la biodiversidad. Los desarrolladores de software (línea naranja) y los usuarios finales (línea azul) interactúan iterativamente (intersecciones de líneas) y se involucran más intensamente (bucles) o intercambian el liderazgo de las interacciones (líneas más gruesas) durante el proceso, mostrando más flexibilidad en cómo y cuándo se involucra a los usuarios finales, en comparación con los marcos ágiles típicos. Además, encontramos que solo ciertos elementos especificados en las metodologías típicas de desarrollo ágil de software eran necesarios, especialmente aquellos relacionados con la inclusión regular de los usuarios y la comunicación con ellos (Pasos B, D, G). Por el contrario, no fue necesario seguir un marco ágil formalizado (como Scrum, con iteraciones (*sprints*) concertados para el desarrollo de tareas particulares).

Las ramas en el Paso H representan cómo el software puede tener utilidad para usuarios adicionales al ser incluido en una ronda posterior de desarrollo.

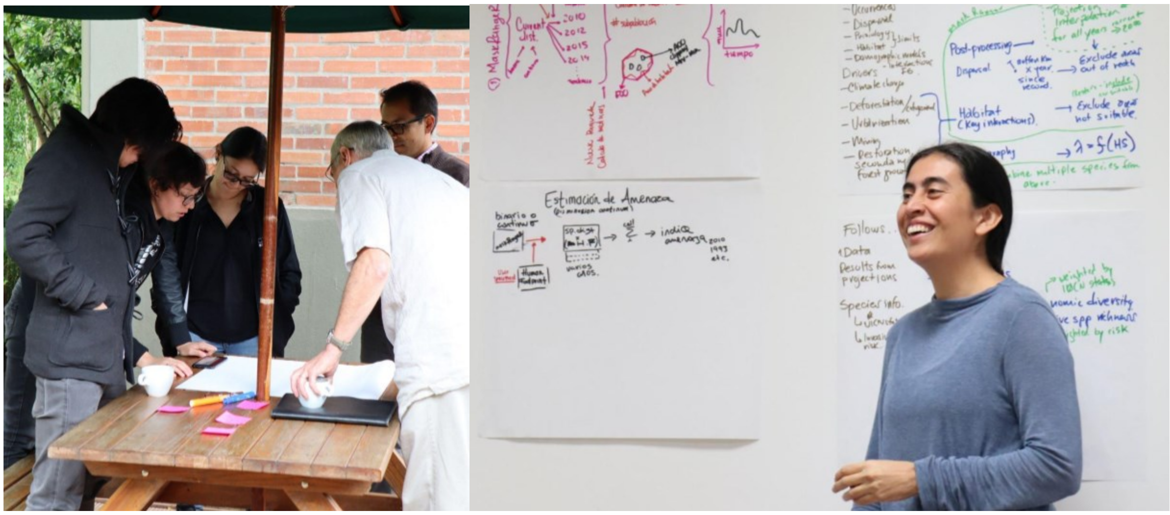

Fig. 2. Ejercicio de visualización de usuarios (izquierda) y discusión (derecha) durante un taller de consulta a usuarios en Bogotá, Colombia en 2019.
